# Predictive models of Alzheimer’s disease dementia risk in older adults with mild cognitive impairment: a systematic review and critical appraisal

**DOI:** 10.1186/s12877-024-05044-8

**Published:** 2024-06-19

**Authors:** Xiaotong Wang, Shi Zhou, Niansi Ye, Yucan Li, Pengjun Zhou, Gao Chen, Hui Hu

**Affiliations:** 1 College of Nursing, Hubei University of Chinese Medicine, Wuhan, China; 2 Engineering Research Center of TCM Protection Technology and New Product Development for the Elderly Brain Health, Ministry of Education, Wuhan, China; 3 Hubei Shizhen Laboratory, Wuhan, China

**Keywords:** Mild cognitive impairment, Elderly, Alzheimer’s disease, Dementia, Predictive model, Systematic review

## Abstract

**Background:**

Mild cognitive impairment has received widespread attention as a high-risk population for Alzheimer’s disease, and many studies have developed or validated predictive models to assess it. However, the performance of the model development remains unknown.

**Objective:**

The objective of this review was to provide an overview of prediction models for the risk of Alzheimer’s disease dementia in older adults with mild cognitive impairment.

**Method:**

PubMed, EMBASE, Web of Science, and MEDLINE were systematically searched up to October 19, 2023. We included cohort studies in which risk prediction models for Alzheimer’s disease dementia in older adults with mild cognitive impairment were developed or validated. The Predictive Model Risk of Bias Assessment Tool (PROBAST) was employed to assess model bias and applicability. Random-effects models combined model AUCs and calculated (approximate) 95% prediction intervals for estimations. Heterogeneity across studies was evaluated using the *I*^*2*^ statistic, and subgroup analyses were conducted to investigate sources of heterogeneity. Additionally, funnel plot analysis was utilized to identify publication bias.

**Results:**

The analysis included 16 studies involving 9290 participants. Frequency analysis of predictors showed that 14 appeared at least twice and more, with age, functional activities questionnaire, and Mini-mental State Examination scores of cognitive functioning being the most common predictors. From the studies, only two models were externally validated. Eleven studies ultimately used machine learning, and four used traditional modelling methods. However, we found that in many of the studies, there were problems with insufficient sample sizes, missing important methodological information, lack of model presentation, and all of the models were rated as having a high or unclear risk of bias. The average AUC of the 15 best-developed predictive models was 0.87 (95% CI: 0.83, 0.90).

**Discussion:**

Most published predictive modelling studies are deficient in rigour, resulting in a high risk of bias. Upcoming research should concentrate on enhancing methodological rigour and conducting external validation of models predicting Alzheimer’s disease dementia. We also emphasize the importance of following the scientific method and transparent reporting to improve the accuracy, generalizability and reproducibility of study results.

**Registration:**

This systematic review was registered in PROSPERO (Registration ID: CRD42023468780).

**Supplementary Information:**

The online version contains supplementary material available at 10.1186/s12877-024-05044-8.

## Introduction

According to the WHO, more than 55 million people worldwide (8.1% of women and 5.4% of men over 65) are currently estimated to be living with dementia. This number is estimated to increase to 78 million by 2030 and 139 million by 2050 [1].So the World Health Organization approved “the Global Action Plan on the Public Health Response to Dementia 2017–2025” at the World Health Assembly in May 2017, which proposes strategies for the prevention and treatment of dementia and provides guidance on improving the quality of life of people living with dementia, their families and caregivers [2]. Alzheimer’s disease (AD) is the most common cause of dementia, accounting for 60–80% of dementia. It has been reported that every patient with AD dementia experiences mild cognitive impairment (MCI), a stage considered to be the transition between normal ageing and dementia. Statistics show that the prevalence of MCI is about 8% in people aged 65 to 69 years, rising to 15% in people aged 70 to 79 years, 25% in people aged 80 to 84 years, and 37% in people aged 85 years and older [3], however, because the current diagnostic and treatment model of cognitive impairment in the elderly has not yet been perfected, coupled with the lack of awareness of the importance of treatment among patients and their families as well as uncertainty about the effectiveness of MCI treatment [4], Existing statistics may significantly underestimate the actual prevalence of MCI in older adults, and these factors also highlight the challenges faced in early screening and intervention services for AD dementia. It has been suggested that without intervention at the MCI stage, about 15% of people with MCI will develop AD dementia after two years [5]. However, effective intervention at this stage can delay cognitive decline [6]. In the face of the challenges of global ageing, screening and treatment of older adults with MCI should receive more attention, and dementia risk prediction is crucial for identifying at-risk populations.

Several studies have indicated that risk prediction models can assist healthcare professionals in identifying patients who are at high risk of cognitive decline. For instance, Wang et al. [7]. developed a predictive model to assess the risk of MCI in normal community-dwelling older adults. Another meta-study by Gopisankar et al. [8]. analyzed risk factors for MCI in Chinese older adults and developed a new hybrid model by updating and evaluating three existing models and applying a deep neural network analysis. This new model demonstrated higher predictive performance in assessing the incidence of dementia. An et al. [9]. conducted a meta-analysis and identified critical indicators of objectively measured cognitive impairment in individuals who have reported experiencing subjective cognitive deterioration. They also developed risk prediction models under two scenarios to identify individuals more likely to experience clinical progression.

However, the prediction models themselves have generated some controversial discussions, such as a meta-analysis by Huang et al. [10] to assess the predictive performance of multivariable prediction models for cognitive decline in older adults, which showed that the usefulness of the models was not very high. Li et al. [11] found that the prediction of individual disease risk varied significantly between different types of machine learning and statistical models with almost the same level of discrimination. However, the different views generated by the above studies are mainly caused by the significant differences in the existing models regarding data sources, sample sizes, and data processing and analysis methods. At present, there is yet to be a consensus on the most effective model. Considering that filling this research gap will be the key to predicting the progression of the disease in older adults with MCI and the implementation of timely and effective diagnosis and treatment, the present study will comprehensively analyze the published dementia-related risk prediction models.

This systematic review and critical evaluation was carried out to: 1) Provide a comprehensive summary of the best-performing multivariable predictive models in all current studies,2) Summarise models relevant data and methodological issues in model development and validation for 16 studies, and 3) Explore whether machine learning and traditional modelling approaches may affect model performance. The outcomes of this study aim to enhance the dependability and precision of AD dementia risk prediction models, thereby informing future efforts in predictive modeling and validation.

## Methods

This study strictly followed the CHARMS checklist for systematically evaluating predictive modelling studies [12]. PROBAST [13, 14] was used to assess the bias and applicability of the predictive modelling studies. We used various methods to obtain estimates and confidence intervals for each study’s optimal model performance measures. For data extraction, we prioritized the performance statistics that were derived using the most convincing validation methods (in increasing order of confidence: external validation, i.e., evaluation in independent populations, internal validation such as Bootstrap validation, cross-validation, random training-test splits and time splits, and the same data as in the development process). Performance statistics (in increasing order of confidence: external validation, i.e., evaluation in an independent population; internal validation, such as Bootstrap validation, cross-validation, randomized training-test splits and time splits, and evaluation with the same data as in the development process) [15], and finally we merged and summarized the data.

### Literature search

Our comprehensive search across PubMed, EMBASE, Web of Science (WOS), and MEDLINE, spanning from each database’s start to October 19, 2023, focused on identifying models designed to estimate the likelihood of AD dementia in individuals 60 years and above who have MCI. Furthermore, we retrieve by using search filters that recognize predictive modelling studies [16]. The filters have been validated to have high sensitivity in retrieving clinical prediction model studies. A comprehensive inventory of the search terms utilized is available in Appendix File 1.

### Eligibility criteria

This analysis encompassed all primary research that either created or confirmed multivariable prediction models (with a minimum of two predictors). A thorough delineation of the study’s population, the primary model being evaluated, the model used for comparison, the outcomes, the time frame, and the context (PICOTS) is depicted in Table 1.

The literature inclusion criteria were as follows:

1. Study population: older adults with MCI with a mean age of 60 years or older.
2. Study content: Studies on risk prediction models for the progression of MCI to AD dementia.
3. Study type: cross-sectional surveys, case-control studies, and cohort studies.
4. Outcome metrics: compliance with the diagnostic criteria for AD(includes the NINCDS-ADRDA [17]diagnostic criteria, any version“Diagnostic and Statistical Manual of Mental Disorders” [18], NIA [19], IWG [20], standardized neuropsychological tests and clinician’s diagnosis or a combination of these criteria.

Literature exclusion criteria were as follows:

1. Studies on specific populations, such as some specific diseases (e.g., organic diseases such as stroke, epilepsy, and Parkinson’s).
2. The number of predictors was less than 2.
3. Duplicate publications.
4. Unofficial publications such as conference abstracts, academic papers, and so on.
5. The language of the article needed to be English.

**Table 1:** Selection criteria of predictive modelling studies in PICOTS format

PICOTS	Explanation
**P**articipants	Older adults with mild cognitive impairment
**I**ndex model	All available prognostic models
**C**omparator model	Not applicable
**O**utcome	diagnosis of AD dementia
**T**iming	Any time interval
**S**etting	Models to be used in old people with mild cognitive impairement to predict risk of development of AD dementia in the future, and inform targeted screening and/or primary prevention

### Screening process

Screening was performed using Endnote X9 software. Initially, two independent reviewers (XW, PZ) screened titles and abstracts for predictive modelling studies based on inclusion and exclusion criteria, with a third reviewer (NY) participating when necessary. After consensus was reached, the full-text literature was independently searched and screened by two reviewers (XW, PZ); in addition, we conducted a manual examination of the reference lists in the selected studies to identify additional studies that might be pertinent [21].

### Data extraction

In this scientific investigation, the data extraction was carried out independently by two researchers, XW and PZ. Use CHARMS [12] to design standardized data extraction forms. The critical information extracted followed the principles of PICOTS, i.e., number of subjects included, data source, predictors (e.g., patient characteristics, imaging or biological markers), model status (e.g., performance, modelling status, and model presentation), and outcome metrics (e.g., measurement tools for AD dementia and duration of follow-up). In addition, information including author names, year of publication, type of study, and statistical information (e.g., treatment of missing data and selection of predictors, treatment of continuous variables) was also collected. Finally, we calculated the minimum sample size by pmsampsize package in R. We reviewed the supplementary material of the articles to ensure that all information about the articles could be extracted in its entirety. (see Appendix Table 6).

In order to analyze the predictive ability of each model, this study intends to analyze the following metrics: Confusion Matrix, Accuracy, Sensitivity, Specificity, Precision, F1 Score, AUC, DCA to evaluate the clinical applicability of the model were extracted.1) Discrimination, which refers to the model’s ability to discriminate between people with AD dementia and those without AD correctly, is often measured by the Consistency Statistics (C-index) and the AUC. The AUC is a measure of the accuracy of the model, the closer the AUC is to 1, the better the diagnostic effectiveness of the model [11]; 2) Calibration, the degree of accuracy of probability prediction is called “calibration degree”, is a way to measure the size of the difference between the probability predicted by the algorithm and the actual result, also called consistency, goodness-of-fit, mainly through the Hosmer-Lemeshow test and the goodness-of-fit curve evaluation [21]; 3)Clinical validity evaluation metrics, DCA, this approach serves to assess clinical predictive models, diagnostic examinations, and molecular markers. It aligns with the practical demands of clinical decision-making processes, often being more prevalent in external validations [22]. In addition to the above several conventional metrics, include the Confusion Matrix, Accuracy, Sensitivity, Specificity, F1-Score, and Brier Score [23]. (see Appendix Table 7)

If inconsistencies are found during the data extraction process, NY will adjudicate these inconsistencies. Our study was guided by a systematic review and meta-analysis of preferred reporting items (TRIPOD-SRMA). For a complete list of search terms used.

### Risk of bias assessment

This review applies the Risk of Bias Assessment Tool (PROBAST) [14] to assess the risk of bias (ROB) and the applicability of prediction models. PROBAST consists of four domains: participants, predictors, outcomes and analysis. Each question can be answered as “yes”, “probably yes”, “probably no”, “no”, or “no information”, As long as one of the domains is answered “no” or “probably not”, the domain is considered high risk; as long as the question in each domain is answered “yes” or “probably yes”, it will be defined as low risk. The overall ROB was deemed low when each domain consistently exhibited a low ROB. Conversely, the ROB was categorized as unclear, in cases where one or several domains exhibit an uncertain ROB, while the remaining domains are assessed as low in ROB. The applicability evaluation was similar to that of ROB, but only the first three domains were used to evaluate the applicability of the predictive model. The first two researchers (XW and PZ) assessed independently, and finally, the third reviewer, NY, made the judgment.

### Statistical analysis

We used the R version 4.3.1 for the meta-analysis. Different from the conventional Meta-analysis, due to the significant heterogeneity of the predictive models, we directly used the random effects model [13, 22], the AUC of the models and the calculated (approximate) 95% prediction intervals were combined and estimated, heterogeneity was quantified using the *I*^*2*^ statistic, where *p* < 0.05 and *I*^*2*^ > 50% signify statistically significant heterogeneity [23]. This statistic indicates the extent of variation across studies attributable to heterogeneity. To further analyze this variation, we divided the studies into two subgroups: those using machine learning modelling and those employing traditional modeling. Additionally, we utilized a funnel plot to illustrate the risk of bias.

## Results

### Selection process

**Fig. 1:**
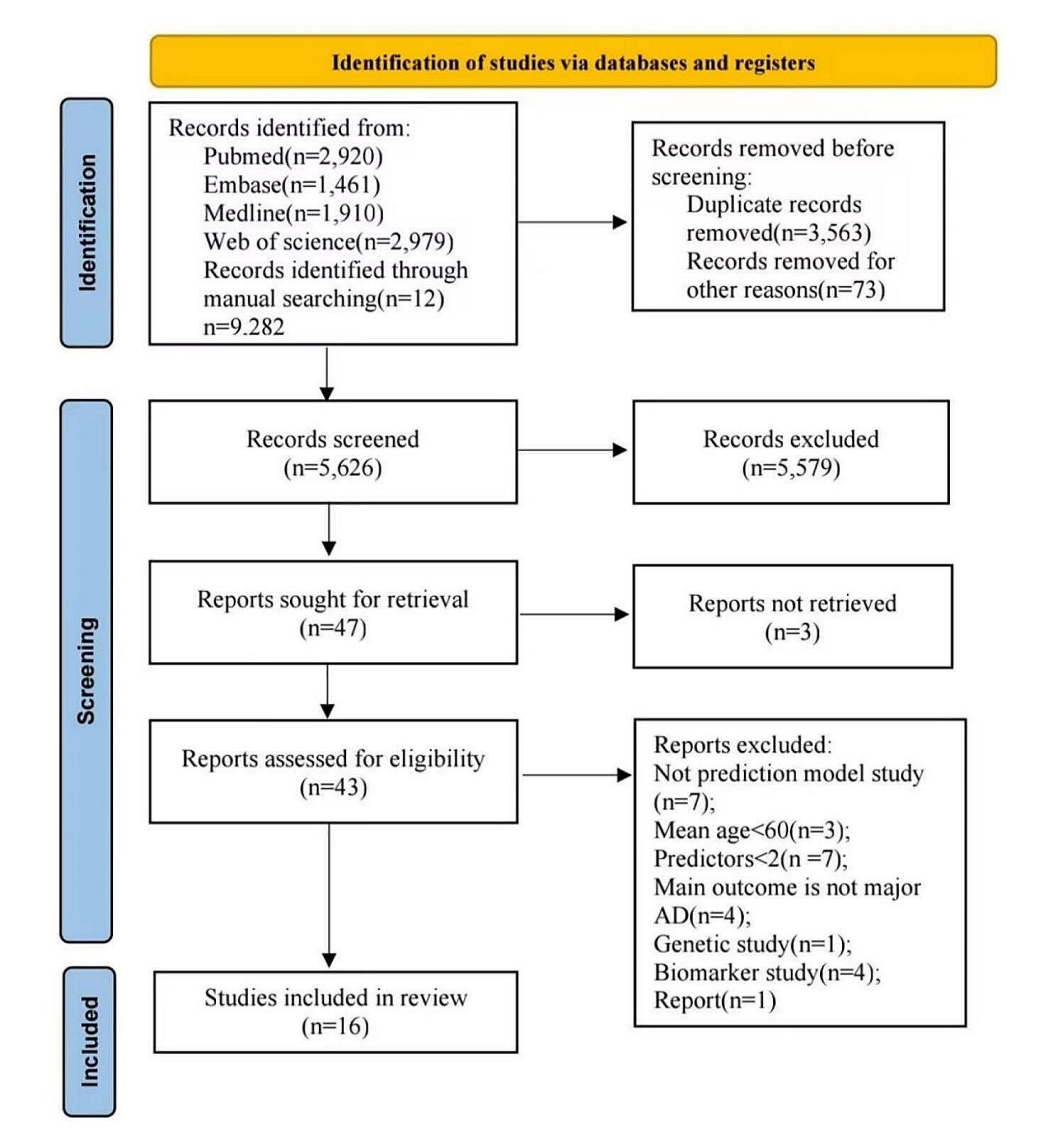
Systematic reviews and Meta-Analyses (PRISMA) flowchart of literature searching and selection

The PRISMA flowchart illustrates our process for searching and selecting literature. In our search, we gathered a total of 3,337 potentially relevant records, sourced from PubMed (2,920 records), EMBASE (1,461 records), Medline (1,910 records), Web of Science (2,979 records), and manual searches (12 records). We removed 3,563 records identified as duplicates, leaving 5,626 unique records for initial review based on titles and abstracts. Of these, 5,579 were subsequently excluded from title and abstract evaluation. Ultimately, we thoroughly reviewed 43 full-text articles, and 16 of these met our criteria for inclusion in this study. The literature screening process is shown in Fig. 1.

### Summary of findings

#### Study designs and population

All prediction models (*n* = 16) were development models. The majority (*n* = 13) were retrospective cohort studies, and three [24–26] were prospective cohort studies. One study [24] was from a medical examination centre. One study [25] was from a memory clinic. One study [26] was recruited from a community-based primary health care centre, two [27, 28] were multicenter retrospective cohort studies, and 11 studies [29–39] were from the ADNI database in North America. In addition, there was one study [29] from the NACC (The National Alzheimer’s Coordinating Center) in the United States, two studies [24, 26] from China, and two other studies from Korea [25] and Spain [30] respectively. In our prediction modelling, the populations studied varied in size, ranging from 102 to 2,611 individuals, and the prevalence of MCI progressing to AD dementia ranged from 15.03 to 52.22%. Detailed characteristics are shown in Appendix Table 6.

#### Predictors

**Fig. 2:**
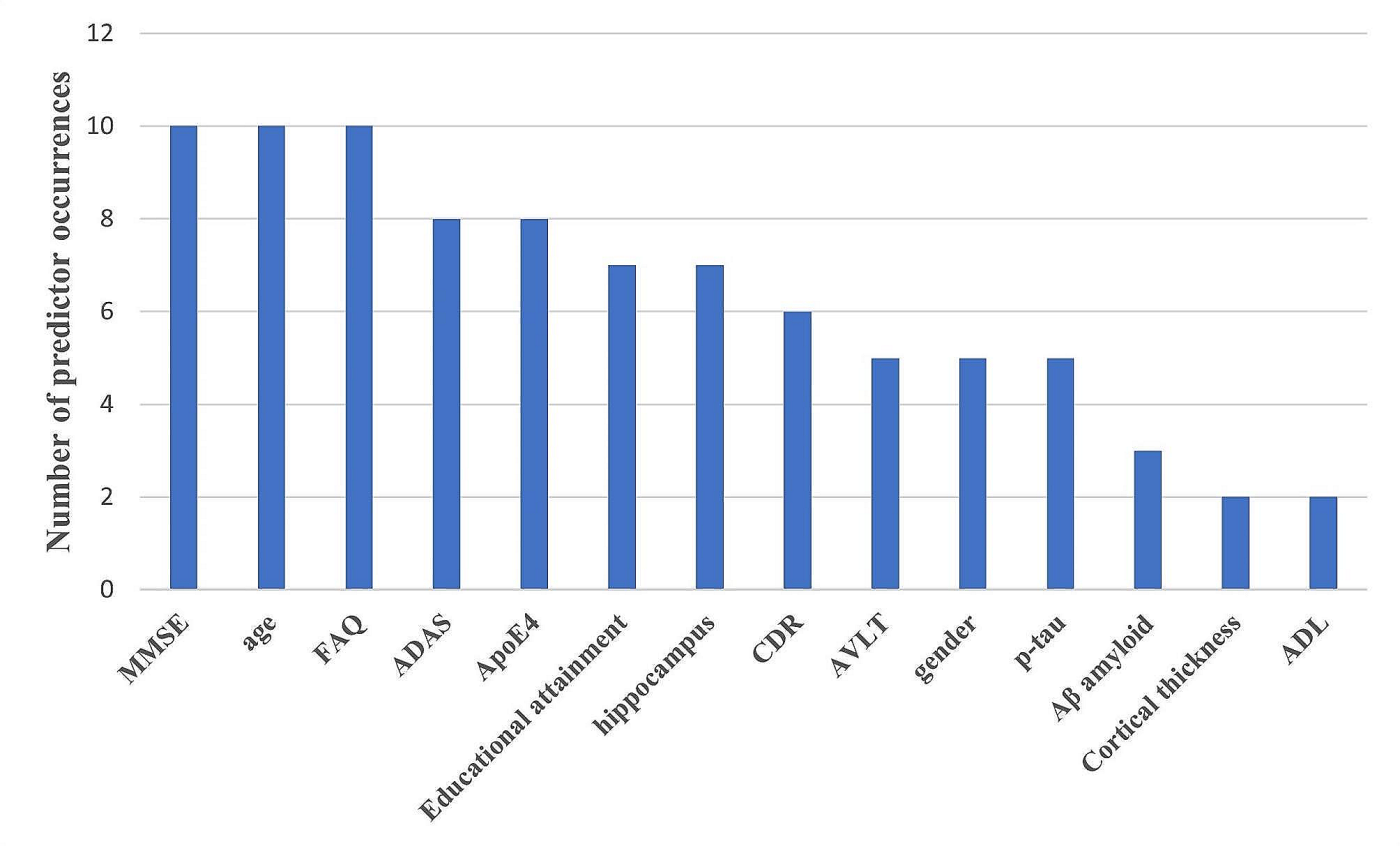
: An overview of the most commonly used predictors in AD dementia risk prediction models. *MMSE: Mini-Mental State Examination; FAQ: Functional Activities Questionnaire; ADAS, Alzheimer’s disease assessment scale-cognitive subscale; APOE4, Apolipoprotein E 4 allele; p-tau: Highly phosphorylated tau protein; CDR, Clinical dementia rating; ADL: Activity of daily living

We identified more than 400 candidate predictors and 94 final variables in our predictive model, divided into four main types: demographic characteristics, health-related risk factors, cognitive scores, and various biomarkers. The following 16 predictors were used at least twice as predictor variables in the model: MMSE, age, FAQ, ADAS, ApoE4, education level, hippocampal volume, CDR, AVLT, gender, p-tau, Aβ amyloid, cortical thickness, and ADL, age (*n* = 10, 62%), MMSE (*n* = 10, 62%), and FAQ (*n* = 62, 14%) were the most common predictors. (See Fig. 2.)

#### Missing data and continuous variables

Three studies used an entire case study approach [24, 27, 31], one study used median imputation [32], one study used machine learning for imputation [39], one study [29] directly deleted missing data, and the remaining ten studies [25, 26, 30, 31, 34–38]did not indicate how the missing values were handled. Four studies [24, 26, 30, 32] converted continuous variables to categorical variables, six studies [25, 27, 28, 36–38] retained continuous variables, and the remaining five studies [29, 31, 33–35, 39] did not indicate how the variable transformation was performed. Regarding the choice of variable screening methods, two models [35, 39] chose LASSO to screen variables, two studies [30, 38] chose RF for screening, and the other studies (*n* = 10) chose COX regression, complete subset regression, and MRMR, in addition to one study [28] that did not specify the screening method.

#### Modelling method and follow-up duration

Most of the forecasting models (*n* = 11) [24–26, 28, 30, 31, 33–39] were developed using machine learning, two studies [27, 31] modeled Cox proportional risk regression models, two studies [29, 32]combined a mixture of modeling approaches for hybrid modeling, and one study [28] did not indicate the model type. The forecast period was 2–7 years, most studies [25–28, 31, 32, 35–39] (*n* = 9) predicted the AD dementia time for medium-term forecast (3–5 years), two models [24, 34] focused on the short-term forecast (1–2 years), and 3 models [29, 30, 32] were for the long term forecast (5–10 years).

#### Model performance and validation

Two models have been externally validated [32, 35], and the rest have only been internally validated. Among the internally validated models, one model [38]used random partitioning, one model [31] used bootstrapping, and eight models [24–27, 30, 34–37] used cross-validation, of which three models [28, 32, 37]used hierarchically nested cross-validation, and two models [29, 33] used combinatorial methods. Regarding model performance, all models reported discrimination except for one model [39], which did not provide discrimination. Fifteen models had an AUC greater than 0.70, and one model [29] had an AUC ranging from 0.50 ?∼ 0.69. In addition, eight models [25, 27, 28, 30, 31, 34, 36–38] reported calibration or predictive model fit goodness-of-fit curves, and all reported models were calibrated. The sensitivity of the models was 0.62 ?∼ 1, and the specificity was 0.69 ?∼ 0.98. Accuracy ranged from 0.50 to 0.99%. (See appendix Table 7)

#### Model presentation

Five models [25, 28, 30, 31, 35, 39] were presented as scoring systems, one model [34] was presented as an equation formula, one model [38] was presented as a graphical scoring method, one model [27] was presented as a web-based calculator and an app, one model [32] was presented as a column-line graph, and the other six models [24, 26, 29, 33, 36, 37] were not reported.

### Risk of bias and applicability

Fourteen models were rated high, and two [25, 34] had an unclear ROB (see Appendix Tables 8 and 9). We found a model with a low risk of bias, but without external validation [29]. Therefore, we classified them as high ROB. Two models [30, 32] were judged to have high ROB in the participant, mainly because the study population did not represent the model’s target population. Most models were judged to have unclear ROB in the outcome, mainly due to some interference between the outcome measures and perhaps the predictors. Four models [24, 27, 28, 37] had high ROBs in the analysis domain related to insufficient sample size, and nine models [25, 26, 29, 31, 33, 35, 37–39] rated as unclear were mainly related to the unclear on the handling of variables.

Regarding applicability, five models [31, 33, 36, 37, 39] were rated as having an unclear concern of applicability in the domain of participants. Two models [29, 30] were highly biased in the outcome domain as long as they were related to a mismatch between the outcome indicator and the systematically evaluated question. This implies that the settings or participant demographics in these predictive modeling studies might not align perfectly with the context of our research question. Overall, most of the model (*n* = 14, 85%) had a high ROB, and about half of the models (*n* = 7, 43%) had a concern of applicability that was unclear or high.

### Meta-analysis of validation models

**Fig. 3:**
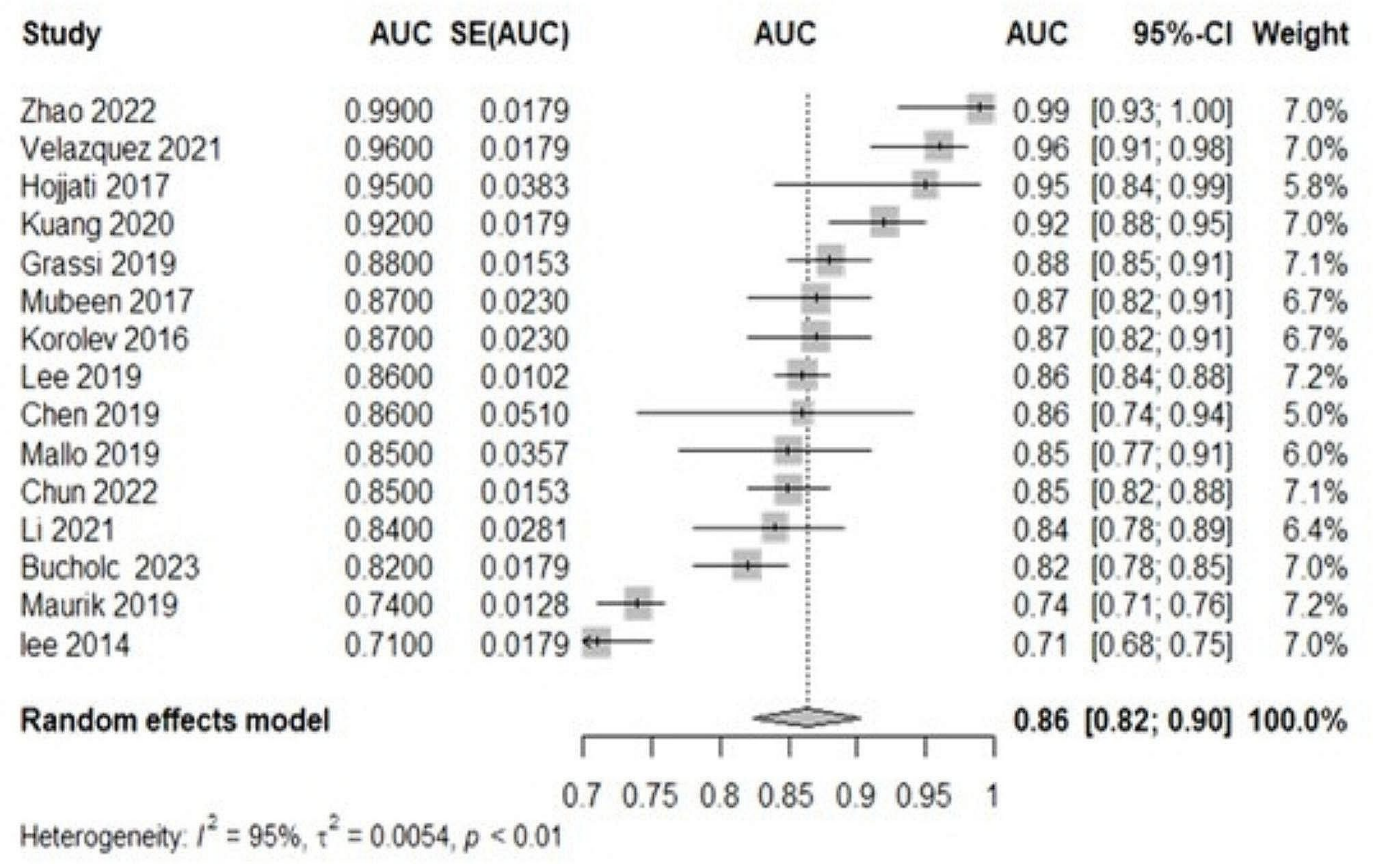
Forest plot of meta-analysis of pooled AUC estimates for 15 validation models. *95% CI, 95% confidence interval; ML: machine learning

We performed a meta-analysis including 15 development models reporting AUC and their 95% confidence intervals (95% CI). The Li et al. [39] study was excluded due to missing AUC. Pooled analysis of the 15 studies showed that we combined all AUCs using a random-effects model, with an AUC of 0.86 (95% CI: 0.82, 0.90) and an *I*^*2*^ of 95% (*p* < 0.01) (Fig. 3.), suggesting high heterogeneity.

**Fig. 4:**
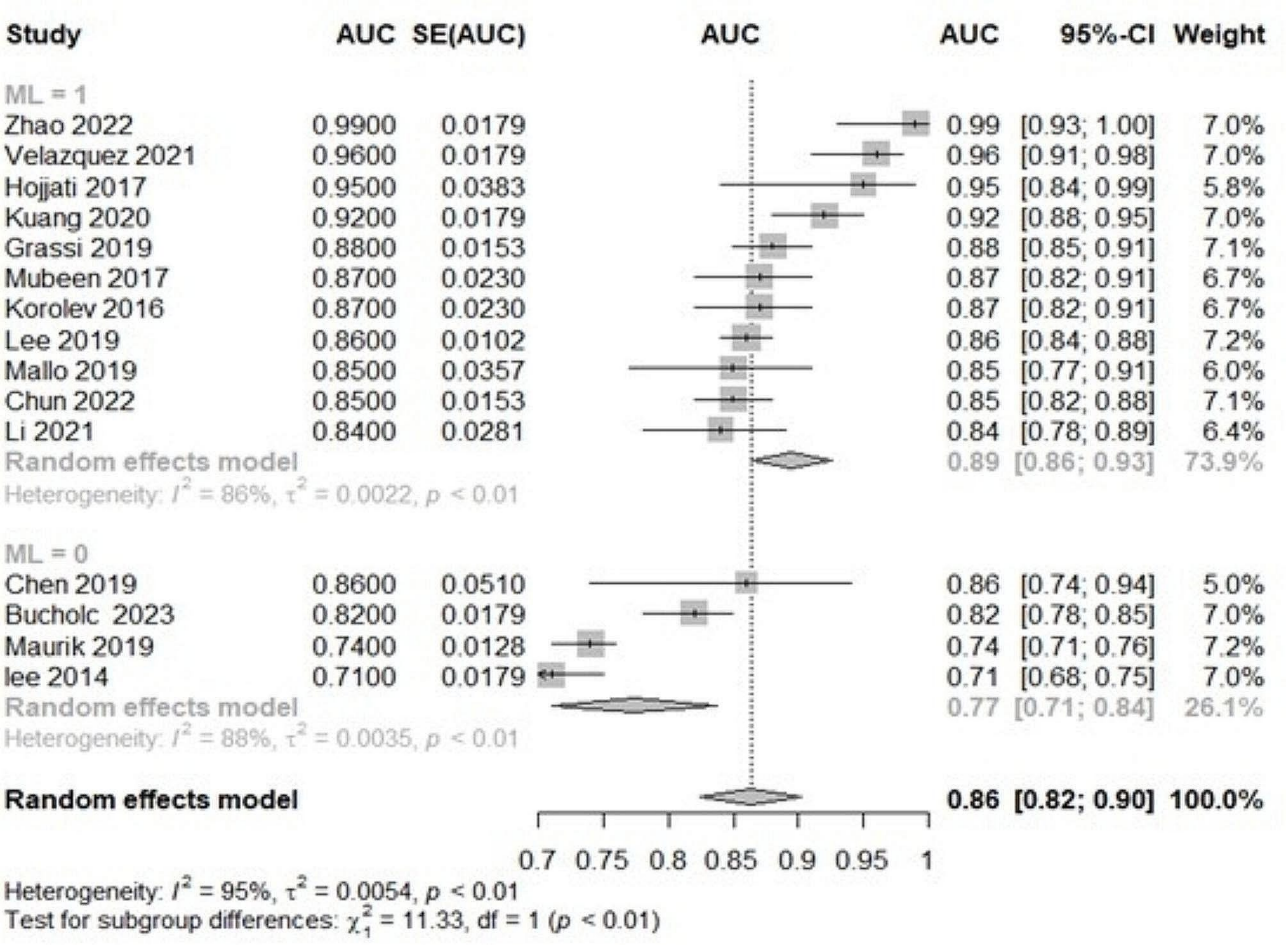
Forest plot subgroup analysis of pooled AUC estimates for 15 validation models. *95% CI, 95% confidence interval; ML: machine learning

In addition, we conducted subgroup analyses by grouping the traditional regression model, and ML showed that the effect size of the traditional regression model subgroup (0.89; 95% CI: 0.86,0.93) was more significant than that of the traditional regression model subgroup (0.77; 95% CI: 0.71,0.74) (Fig. 4.), and the results suggest that different modelling may be a potential heterogeneity of study results Reason. Publication bias was evaluated using funnel plots (see Appendix Fig. 3). The distribution of the scatter plot appeared largely symmetric, indicating an absence of notable publication bias in the prediction models analyzed.

## Discussion

### Principal findings

This research offers a comprehensive analysis of predictive models aimed at determining the risk of AD dementia in elderly with MCI, examining 16 different models. These models, formulated in both community and clinical environments, primarily target the elderly population, including those attending memory clinics. Notably, Five studies opted for Random Forest (RF) as the primary modeling tool during model construction. This choice was primarily due to RF’s ability to robustly handle high-dimensional data and demonstrate strong generalization capabilities [40]. Additionally, two studies employed the Cox Proportional Hazards Model (COX), exhibiting exceptional survival analysis performance [28]. Researchers also favored fusion models as they combine the strengths of multiple models, significantly enhancing prediction accuracy [29, 32]. Support Vector Machine (SVM) was often used in some studies to classify data [33]. Cyclic Neural Networks (RNN) demonstrated unique advantages in capturing time dependencies, while Artificial Neural Networks (ANN) stood out for their powerful learning capabilities [41]. When dealing with large-scale datasets, eXtreme Gradient Boosting (XGBoost) exhibited efficiency, and the Variational Bayes (VB) method provided valuable uncertainty estimations [25]. Each modeling approach possesses unique advantages and different application scenarios.

Then, we conducted a subgroup analysis of ML versus traditional modelling approaches and found that ML algorithms were more effective than traditional regression models in outcome prediction. However, it has been argued [42] that the reliance on large amounts of data for machine-learning approaches may limit their effectiveness in small sample datasets. Reinke et al. [43] compared classical and ML approaches to develop dementia risk prediction models and found that ML did not outperform logistic regression, confirming the importance of sample size. This also shows that the data and features determine the upper bound of the predictive model, and the model and algorithm only help the research to keep approaching the upper limit.

Furthermore, by summarizing the most used predictors in prediction models for the stage from MCI to AD dementia, our findings are inconsistent with An et al. [9], mainly due to inconsistencies in the study population. Although the close association of cognitive biomarkers with the pathology of the disease makes them overall superior to epidemiology and neuropsychology, we observed that four of the top five predictors were non-cognitive biomarkers. Only ApoE4 was a cognitive biomarker, which may result from some scholars’ preference to use relatively valid demographic characteristics and neuropsychological scales due to cost considerations as predictor variables. Given this, we suggest that future studies develop more diverse predictive models based on different clinical settings, community environments, and individual circumstances.

Most of the studies were developing new models, and our assessment of them found that the predictive power of the models ranged from moderate to excellent. However, these models consistently have a high or unclear ROB. With three models [26, 28, 31] showing low ROB in all but the analysis, suggesting that the models performed well in terms of study design and data collection but had problems in terms of statistical analysis, commonly such as insufficient sample size, insufficient consideration of model overfitting issues, use of missing data and unclear treatment of continuous variables [44], it is likely to result in good performance on the training set but poor performance on the test set or in real-world applications. This means that while the model learns the features of the training data well, it cannot generalize to new data effectively, dramatically reducing the model’s usefulness and reliability [45]. Although predictive modelling holds some promise for improving AD dementia prevention and intervention, given the insufficient evidence, it is yet to be possible to recommend any predictive model for widespread use in practice.

### Challenges and opportunities

This comprehensive analysis identified certain methodological shortcomings in the integrated development or validation of predictive models.

First, although many models have been internally validated and calibrated, only a few have been externally validated. It is worth noting that predictive models usually outperform external validation in model development data, but external validation is more convincing than internal validation [46]. Therefore, to ensure the generalizability of the models, we emphasize the importance of using different datasets as much as possible to validate the existing model’s performance. In validation studies, we need to verify that the model’s performance (discrimination and calibration, especially discrimination) on new data is close to the performance on the data on which it was developed [47], and the assessment of model usefulness requires a clinical judgment; furthermore, in machine learning the performance of a model may undergo the concept drift, over time, and thus continuous validation and updating of the predictive model is necessary to ensure applicability to new populations.

In addition, we found that about half of the models suffered from direct deletion of missing values and incomplete reporting. Failure to treat missing data appropriately usually leads to biased effect estimates because missing data can distort the performance of a predictive model if it is correlated with other variables [48]. Missing value imputation methods are categorized into deletion, simple imputation, multiple imputation, and algorithmic imputation, while multiple imputation [49] and Miss Forest [50] are currently the more recommended methods. About half of the studies transformed continuous variables into binary or multi-class classification. However, many researchers have hotly debated the treatment of continuous-type variables. From a statistical point of view, downgrading continuous variables to categorical variables, especially binary classification is highly likely to result in the loss of data information and reduced prediction performance. However, from the clinical point of view, it is easier to quickly determine the outcome of the patients [51–53]. Therefore, which method should be taken should be considered according to the purpose of the study, the method used, and the data.

In addition, we note that different methods were used to screen the model’s predictor variables. If the number of variables is too high, the model may overfit the training data, decreasing predictive performance on new data, and too few may lead to poor model performance [54]. Thus, it is essential to ensure that the model captures the key features of the data while keeping the model concise. There are three general categories of variable selection methods: filter, wrapper, and embedding [55]. The most appropriate method for selecting predictors still needs to be discovered. However, for data with many features (or data that exhibit multicollinearity), it is expected to use regularized regression (often also known as penalized models or shrinkage methods) to impose restrictions and thus reduce the occurrence of overfitting. In addition, the sample size is also closely related to the variables. In addition to the traditional 10-EPV estimation method, sample size calculation tools have been developed to estimate the sample size of clinical prediction models [56].

Furthermore, the practical application of risk models should take into account their cost-effectiveness. Typically, models that incorporate high-cost predictors tend to exhibit greater predictive accuracy compared to solely depending on the judgment of clinicians [57, 58]. However, model feasibility and cost constraints can limit model use, particularly in primary care [59]. Model simplicity and measurement reliability are essential for developing clinically useful prognostic models. The current study shows that clinical judgment frequently demonstrates comparable or superior performance compared to predictive models, and some predictors may instead limit the use of these models due to their invasive nature, the high cost of the tests, and the non-routine nature of the tests [60]. Therefore, we suggest that future studies need to consult with clinical experts on the one hand for their opinions and insights, including the interpretability and applicability of these variables in actual clinical settings, in addition, continuously updating current models by mining predictors with more vital incremental value (exploring non-cognitive biomarkers with modifiable properties and more common biomarkers) to identify patients at high risk of AD dementia more effectively.

Finally, we found that the included studies needed more model presentation, incomplete regression equations and lack of clarity about the intended use of the model used. An inadequate presentation of research studies not only represents a significant squandering of research resources but also obstructs future activities such as validation, updating, recalibration, and providing direction for clinical practice. In terms of model presentation, in addition to providing complete model equations, there are many forms, such as scoring systems, column-line graphs, web calculators, and apps. In addition, Bonnett et al. [61] point out that even if models do not perform very well, they may still be of clinical utility. Therefore, indicating that the specific intended use of a predictive model (i.e., when or where they will be used in an investigation and for whom they will be used) may be equally relevant may be helpful.

### Advantages and limitations

This study represents the first comprehensive and integrated assessment of AD dementia risk prediction models for elderly with MCI. Critical features were assembled through an extensive literature search, meticulous screening, and standardized data extraction, thus providing valuable research information for primary healthcare systems and clinical healthcare professionals. This approach lays the foundation for more effective construction and external validation of future predictive models. Furthermore, this study conducted a risk of bias (ROB) assessment and applicability assessment of the prediction model using the PROBAST tool, alongside subgroup and bias analyses, constituting another significant strength in our study.

However, this review is subject to certain limitations. First, despite identifying multiple models that predict conditions in similar populations, a comprehensive meta-analysis of the discrimination and calibration of these models was not feasible due to the lack of detailed calibration reports. Second, the “best model” selection might have overlooked some essential information due to incomplete data extraction. Additionally, despite using random-effects models, there was still a high heterogeneity among the included studies, mainly because there were also unadjustable differences between the patient environments used for study design, measurement methods, and patient characteristics. Heterogeneity is also usually accepted in Meta-analyses of predictive models, but future subgroup analyses of its variability in relevant settings and populations by meta are necessary.

### Future research

To help make clinical decisions founded on the most robust available evidence and to identify the most effective models advocated or utilized for predicting the risk of AD dementia in older adults with MCI. However, we are unable to recommend any specific model due to several reasons. Firstly, nearly all predictive models reviewed exhibited unclear or high risk of bias (ROBs), and the included developmental models required further external validation. Additionally, the significant heterogeneity among the included models, the use of non-standardized statistical analysis methods, incomplete data in model reports, and a lack of analysis regarding the clinical application value all contribute to the challenge of selecting the optimal model. Finally, while the average AUC of our best-developed model has achieved 0.87, some even exceeding 0.98, these findings may not fully translate into actual medical practice [62]. This is because although AUC is a widely utilized metric for evaluating the performance of predictive models, it only partially represents their actual efficacy. Pursuing excessively high values under the receiver operating characteristic AUC maybe over-optimization and potential distortion of the models [63].

Based on these methodological shortcomings, we make the following recommendations. First, models should be externally validated several times in different populations, and sample sizes must be adequately considered. Second, when data are missing, interpolation should be performed using multiple interpolation or machine learning. Third, predictive variables with incremental solid value should be mined based on clinical feasibility and applicability, and preventing overfitting should be emphasized in the predictive model. Fourth, it is clear that medical predictive modelling aims to construct a predictive tool with practical application value. Therefore, when constructing a medical predictive model, it is necessary to start from the practical point of view and give full consideration to the application prospect of the model. At the same time, we also need to recognize the importance of diversified evaluation, as far as possible, sensitivity, specificity, calibration index, net benefit, and DCA for comprehensive evaluation.

## Conclusions

We identified 16 predictive models, most of the researchers reported excellent discrimination in their study. However, for various reasons, the risk of bias in nearly all models was high or unclear. Consequently, this finding implies that the predictive performance of these models might be overestimated, their accuracy in practical application to the target population remains questionable, and currently, we cannot endorse any of these predictive models for clinical practice. Additionally, our exploration of potential predictors, translating evidence into new insights for clinical practice. Future studies on predictive modelling of AD dementia risk in older adults should adhere to methodological guidelines and prioritize practicality and cost-effectiveness in model evaluation, thereby facilitating disease progression identification in older adults with MCI.

### Electronic supplementary material

Below is the link to the electronic supplementary material.

Supplementary Material 1

## Data Availability

All data generated or analyzed during this study are included in the Appendix. The corresponding author can provide the code upon request.

## Abbreviations

AD: Alzheimer’s disease
AUC: Area under curve
ADAS: Alzheimer’s disease assessment scale-cognitive subscale
APOE4: Apolipoprotein E 4 allele
p-tau: Highly phosphorylated tau protein
ADL: Activity of daily living
CDR: Clinical dementia rating
DCA: Decision curve analysis
MCI: Mild cognitive impairment
MMSE: Mini-Mental State Examination
FAQ: Functional Activities Questionnaire
RF: Random forests
ML: Machine learning
LASSO: Least absolute shrinkage and selection operator
MRMR: Max-Relevance and Min-Redundancy
ROB: Risk of bias
