# Supplementary material for: Predictive models of Alzheimer’s disease dementia risk in older adults with mild cognitive impairment: a systematic review and critical appraisal
Source: BMC Geriatr. 2024 Jun 19;24:531. doi: 10.1186/s12877-024-05044-8 (PMC11188292; doi:10.1186/s12877-024-05044-8)
Supplement: Supplementary file 1 — Supplementary Material 1 [file 12877_2024_5044_MOESM1_ESM.docx]

**Supplementary Appendix**

**CONTENTS**

[Table 1: Geersing Search terms included in the filters 1](#_Toc151737701)

[FILE 1: Search Terms and search strategy with full results 2](#_Toc151737702)

[**Table 2: Database(s): Ovid MEDLINE(R) ALL <By October 19,2023>** 2](#_Toc151737703)

[**Table 3: Database(s): Embase <By October 19,2023>** 3](#_Toc151737704)

[**Table 4: Database(s): Pubmed <By October 19,2023>** 4](#_Toc151737705)

[**Table 5: Database(s): Web Of Science <By October 19,2023>** 6](#_Toc151737706)

[Table 6: Characteristic of the Included Studies and model development. 7](#_Toc151737707)

[Table 7: Model Validation and Performance 16](#_Toc151737708)

[Table 8: Assessment of risk of bias and concerns regarding applicability 18](#_Toc151737709)

[Table 9: Risk of bias and applicability assessment for each PROBAST domain 20](#_Toc151737710)

[Fig.1: Prediction model Risk Of Bias assessment Tool (PROBAST) risk of bias assessment for all included models (N =16). 21](#_Toc151737711)

[Fig.2: Prediction model Applicability assessment Tool (PROBAST) risk of bias assessment for all included models (N =16). 21](#_Toc151737712)

[Fig3: Funnel plot of all models included in primary meta-analysis 22](#_Toc151737713)

[Table 10: TRIPOD-SRMA Checklist for reporting systematic reviews of prediction model studies 23](#_Toc151737714)

[Table 11: PRISMA ABSTRACT CHECKLIST 29](#_Toc151737715)

**Table 1: Geersing Search terms included in the filters**

| **Filter** | **Search terms included in the filters** |
| --- | --- |
| **Geersing** | **“stratification” OR “ROC curve” [MeSH] OR “discrimination” OR “discriminate” OR “c-statistic” OR “c statistic” OR “area under the curve” OR “AUC” OR “calibration” OR “indices” OR “algorithm” OR “multivariable”** |

**FILE 1: Search Terms and search strategy with full results**

**Table 2: Database(s): Ovid MEDLINE(R) ALL <By October 19,2023>**

**Search Strategy:**

| **#** | **Searches** | **Results** |
| --- | --- | --- |
| **1** | **SU aged OR AB( older adults or elderly or geriatric or geriatrics or aging or senior or seniors or older people or aged 65 or 65+ )** | **3,263,855** |
| **2** | **SU cognitive dysfunction OR AB ( “Cognitive Dysfunctions” OR “Cognitive Disorder” OR “Cognitive Impairments” OR “Cognitive Impairment” OR “Mild Cognitive Impairment” OR “Cognitive Decline” )** | **79,937** |
| **3** | **(dementia OR AD OR Dementias OR Amentia OR Amentias ).ti,ab.**  **SU dementia OR AB ( AD OR Dementias OR Amentia OR Amentias OR Alzheimer's disease )** | **225,220** |
| **4** | **SU ( “stratification” OR “ROC curve” ) OR AB ( “discrimination” OR “discriminate” OR “c-statistic” OR “c statistic” OR “area under the curve” OR “AUC” OR “calibration” OR “indices” OR “algorithm” OR “multivariable” )** | **655,950** |
| **5** | **(SU “stratification” OR “ROC curve” OR AB “discrimination” OR “discriminate” OR “c-statistic” OR “c statistic” OR “area under the curve” OR “AUC” OR “calibration” OR “indices” OR “algorithm” OR “multivariable”) AND (S1 AND S2 AND S3 AND S4)** | **2,010** |
| **6** | **exp animals/ not humans/** | **1935** |
| **7** | **Limit 13 to English language** | **1910** |

**Table 3: Database(s): Embase <By October 19,2023>**

**Search Strategy:**

| **#** | **Searches** | **Results** |
| --- | --- | --- |
| **1** | **'aged'/exp OR aged** | **5,796,012** |
| **2** | **'older adults':ti,ab,kw OR 'elderly':ti,ab,kw OR 'geriatric':ti,ab,kw OR 'geriatrics':ti,ab,kw OR 'aging':ti,ab,kw OR 'senior':ti,ab,kw OR 'seniors':ti,ab,kw OR 'older people':ti,ab,kw** | **943,876** |
| **4** | **'cognitive dysfunction'/exp OR 'cognitive dysfunction' OR (cognitive AND dysfunction)** | **653,713** |
| **5** | **'older adults':ti,ab,kw OR 'elderly':ti,ab,kw OR 'geriatric':ti,ab,kw OR 'geriatrics':ti,ab,kw OR 'aging':ti,ab,kw OR 'senior':ti,ab,kw OR 'seniors':ti,ab,kw OR 'older people':ti,ab,kw** | **943,876** |
| **6** | **'dementia'/exp OR dementia** | **499,461** |
| **7** | **'ad':ti,ab,kw OR 'dementias':ti,ab,kw OR 'amentia':ti,ab,kw OR 'amentias':ti,ab,kw** | **271,470** |
| **8** | **'stratification'/exp OR 'stratification' OR 'roc curve'/exp OR 'roc curve'** | **382,097** |
|  | **'discrimination':ti,ab,kw OR 'discriminate':ti,ab,kw OR 'c-statistic':ti,ab,kw OR 'c statistic':ti,ab,kw OR 'area under the curve':ti,ab,kw OR 'auc':ti,ab,kw OR 'calibration':ti,ab,kw OR 'indices':ti,ab,kw OR 'algorithm':ti,ab,kw OR 'multivariable'** | **1,394,695** |
| **9** | **#1 OR #2** | **6,135,039** |
| **10** | **#3 OR #4** | **1,639,365** |
| **11** | **#5 OR #6** | **640,189** |
| **12** | **#7 OR #8** | **1,483,403** |
| **13** | **#9 AND #10 AND #11 AND #12** | **14,617** |
| **14** | **#9 AND #10 AND #11 AND #12 AND (/lim AND [english] AND [humans]/lim** | **1461** |

**Table 4: Database(s): Pubmed <By October 19,2023>**

**Search Strategy:**

| **#** | **Searches** | **Results** |
| --- | --- | --- |
| **1** | **Search:Aged[MeSH Terms]** | **3,464,398** |
| **2** | **older adults[Title/Abstract] OR elderly[Title/Abstract] OR geriatric[Title/Abstract] OR  geriatrics[Title/Abstract] OR aging[Title/Abstract] OR senior[Title/Abstract] OR seniors [Title/Abstract] OR older people[Title/Abstract]** | **711,401** |
| **3** | **1 OR 2** | **3,790,457** |
| **4** | **“Cognitive Dysfunction”[MeSH Terms]** | **37,128** |
| **5** | **“Cognitive Dysfunctions”[Title/Abstract] OR “Cognitive Disorder”[Title/Abstract] OR  “Cognitive Impairments”[Title/Abstract] OR “Cognitive Impairment”[Title/Abstract] OR  “Mild Cognitive Impairment”[Title/Abstract] OR “Cognitive Decline”[Title/Abstract]** | **118,622** |
| **6** | **4 OR 5** | **126,424** |
| **7** | **dementia** | **207,257** |
| **8** | **AD OR Dementias OR Amentia OR Amentias OR Alzheimer's disease** | **262,086** |
| **9** | **6 OR 7** | **363,276** |
| **10** | **“stratification” OR “ROC curve”[MeSH Terms]** | **164,141** |
| **11** | **discrimination[Title/Abstract] OR “discriminate”[Title/Abstract] OR “c-statistic”[Title/Abstract] OR”c statistic”[Title/Abstract] OR “area under the curve”[Title/Abstract] OR “AUC”[Title/Abstract] OR “calibration”[Title/Abstract]  OR “indices”[Title/Abstract] OR “algorithm”[Title/Abstract] OR “multivariable”[Title/Abstract]** | **1,036,025** |
| **12** | **7 OR 8** | **1,156,119** |
| **13** | **3 AND 6 AND 9 AND 12** | **2,929** |
| **14** | **exp animals/ not humans/** | **2,924** |
| **16** | **Limit 15 to english language** | **2,920** |

**Table 5: Database(s): Web Of Science <By October 19,2023>**

**Search Strategy:**

| **1** | **TS=(Aged )** | **1,650,693** |
| --- | --- | --- |
| **2** | **TS=(older adults OR elderly OR geriatric OR geriatrics  OR aging OR senior OR seniors OR older people )** | **1,815,814** |
| **3** | **#1 OR #2** | **1,815,814** |
| **4** | **TS=(Cognitive Dysfunction)** | **38,444** |
| **5** | **TS=(“Cognitive Dysfunctions” OR “Cognitive Disorder” OR  “Cognitive Impairments” OR “Cognitive Impairment”  OR “Mild Cognitive Impairment” OR “Cognitive Decline” )** | **99,284** |
| **6** | **#4 OR #5** | **118,801** |
| **7** | **TS=(dementia)** | **98,350** |
| **8** | **TS=(AD OR Dementias OR Amentia OR Amentias OR Alzheimer's disease)** | **232,299** |
| **9** | **#7 OR #8** | **23,299** |
| **10** | **TS=(“stratification” OR “ROC curve” OR “discrimination” OR  “discriminate” OR “c-statistic” OR “c statistic” OR “area under the curve”  OR “AUC” OR “calibration” OR “indices” OR “algorithm” OR “multivariable”)** | **1,560,087** |
| **11** | **#10 AND #9 AND #6 AND #3** | **3,163** |
| **12** | **#11 and Review Article or Early Access or Proceeding Paper #3 and Review Article or Early Access or Proceeding Paper or Editorial Material or Book Chapters or Meeting or Meeting Abstract (Exclude – Document Types) and English (Languages)** | **2,979** |

**Table 6: Characteristic of the Included Studies and model development.**

| **author，year** | **Observed dementia/ total population** | **Sample**  **Size** | **Study (cohort) and** **Follow up duration** | **Study main purpose or result** | **Research**  **Data**  **sources** | **Dementia**  **(outcome)**  **measurement** | **Missing**  **value**  **handling** | **Methods for the inclusion of variables** | **Continuous**  **variables** | **Predictors in final model** | **final model** |
| --- | --- | --- | --- | --- | --- | --- | --- | --- | --- | --- | --- |
| **lee 2014** | **NR/382** | **NR** | **3-year retrospective cohort study** | **14 % low risk scores progressed to AD, 51 % intermediate risk scores and 91 % high risk scores progressed to AD within three years** | **ADNI-1** | **NINCDS-ADRDA** | **Full case study** | **cox proportional-hazards model** | **Keep continuous variables** | **8, gender, patient's attitude towards caregiver (stubbornness/refusal to help), dissociative disorder, basic self-care skills, remembering important appointments and events, Word recall, orientation, clock test** | **Cox proportional hazards regression model** |
| **Grassi 2019** | **197/550** | **361** | **3-year retrospective cohort study** | **Identifying the probability of MCI conversion to AD, and conversion rate of 20–40% from MCI to AD in 3 years** | **ADNIMERGE (subset of ADNI): mainly processed all data since 2004 with missing values and standardized data format, aged between 55 and 90** | **NINCDS-ADRDA** | **Median imputation** | **Fliter, one - hot, Wrapper, LR, RF** | **converted to categorical variables** | **15，age, education, CDR/CDRSB, MMSE, ADAS11, ADAS13, ADASQ4, RAVLT, RAVLT-F, RAVLT-I, RAVLT-L, RAVLT-FP, TMTBT, FAQ, LDT** | **Merge models: LR, NB, L1, and L2 regularized LR or EN, linear SVM, SVM-RBF, and polynomial (SVM-Poly) kernels with Pratt scaling, kNN, multilayer perceptrons with one or two hidden layers, and use full-batch gradient descent or adam algorithms (MLP1-batch, MLP2-batch, MLP1-adam, MLP2-adam), GTB** |
| **Mubeen 2017** | **129/247** | **384** | **Six-month retrospective cohort study** | **Identifying the probability of MCI transformation to sMCI and pMCI** | **ADNI-1** | **NINCDS/ADRDA** | **NR** | **NR** | **NR** | **18, gender, age, education, ApoE4, MMSE, CDR-SB, ADAS13, FAQ, CIR, LHVI, RHVI at baseline and at six months** | **RF** |
| **Maurik 2019** | **1007/2611** | **365** | **5-year Multicenter retrospective cohort study** | **The risk of progression might be higher than 50%, whereas with normal biomarkers, this risk can be far lower than 50%** | **MCI datasets for single- and multi-center cohorts in Europe and North America: European Alzheimer's Disease Medical Information Framework (EMIF-AD; n=883), the Alzheimer's Disease Neuroimaging Initiative (ADNI; n=829), Amsterdam Dementia Cohort (ADC; n=666) and the Swedish BioFINDER study (n=233)** | **NIA-AA** | **Full case study** | **Backwards with old case risk analysis** | **Keep continuous variables** | **7, MMSE, Abeta, p-tau, hippocampal volume, Abeta*p-tau, Abeta*age, p-tau*MMSE** | **Cox proportional hazards regression model** |
| **Bucholc 2023** | **177/1177** | **4489** | **7-year retrospective cohort study** | **Identifying the probability of MCI conversion to AD** | **The National Alzheimer's Coordinating Center Uniform Data Set (NACC-UDS): Recruitment of participants is conducted through referrals from neurologists and community outreach.** | **DSM-IV/NINDS-ADRDA** | **Delete directly** | **BSR** | **NR** | **8, ATL, FAQ, LOGIC MEMORY IIA Delay (LOGIMEM), MMSE, Digit Span Forward (DIGIF) and Digit Span Backward (DIGIB), WAIS-R Numeric Notation (WAIS), Boston Naming Test (BOSTON)** | **A hybrid machine learning model that combines RF, SVM, LR** |
| **Zhao 2022** | **41/105** | **5623** | **2-year prospective cohort study** | **Identifying the probability of MCI conversion to AD** | **All study participants were from communities in Shanghai, China, and were conducted during physical examination at a physical examination center, and the study subjects were 105 elderly adults with MCI aged ≥ 65 years who participated in the physical examination** | **DSM - IV** | **Full case study** | **RFA** | **converted to categorical variables** | **5, ApoE4, the FA value of the left fusiform gyrus, the FA value of the left inferior temporal gyrus, and the FA value of the left parahippocampal gyrus, p-tau** | **RF** |
| **Mallo 2019** | **50/128** | **456** | **6-year retrospective cohort study** | **Identifying the probability of Neuropsychiatric symptoms in conversion from MCI to dementia.** | **Data were collected from 128 people aged 50 years and older in public primary care centres in Santiago de Compostela and Vigo (Galicia, northwestern Spain) between 2 January 2008 and 11 November 2012, with participants completing a baseline neurocognitive assessment and being followed up to 72 months** | **DSM-IV-TR** | **NR** | **RF** | **converted to categorical variables** | **5, age, MMSE, NPI-Q severity score, NPI-Q total pressure score, GDS-15** | **RF** |
| **Lee 2019** | **338/1618** | **335** | **2-year Multicenter longitudinal cohort study** | **Identifying the probability of MCI progressing to AD** | **ADNI** | **NR** | **NR** | **NR** | **NR** | **12, age, gender, years of education, ApoE4, executive function (ADNI-EF), and memory (ADNI-MEM) Aβ1-42, total tau protein (t-tau),**  **ADNI neuropsychological battery using item phosphorylated tau protein (p-tau), hippocampal volume, and entorhinal cortex thickness** | **RNN** |
| **Li 2021** | **79/223** | **352** | **3-year retrospective cohort study** | **Predicting the conversion from MCI to AD in three years according to MCI patient subtype.** | **125 patients with MCI were used in the ADNI-1 dataset and 98 patients with MCI were used in the ADNI-GO/2 dataset** | **NR** | **ML** | **Lasso** | **NR** | **5, SNP, mRNA, hippocampus, inferior parietal lobe, and posterior parietal gyrus** | **variational Bayes approximation with probabilistic multiple kernel learning (VBpMKL) algorithm** |
| **Hojjati 2017** | **18/146** | **NR** | **3-year retrospective cohort study** | **Identifying patients MCI-C and MCI-NC ，** **probability of MCI to AD** | **ADNI** | **NR** | **NR** | **MRMR, Fisher score,**  **chi-square score, Gini score, Kruskal-Wallis test** | **Keep continuous variables** | **5, CDR, MMSE, FAQ, hippocampal volume** | **SVM** |
| **Korolev 2016** | **139/259** | **383** | **3-year retrospective cohort study** | **Identifying patients P-MCI and -N-MCI，** **probability of MCI to AD** | **ADAI-1, developed using only data from patients with the single-domain amnesic subtype of MCI** | **NR** | **NR** | **MRMR** | **Keep continuous variables** | **12, age, gender, education level, no use of anti-Alzheimer's drugs, risk of multiple cerebrovascular diseases, ADAS-Cog, FAQ, Ray Auditory Speech Learning Test (RAVLT), temporoparietal region volume, cortical thickness measurement, A protein involved in vascular processes, immune function and inflammation, and lipid metabolism, ApoE4** | **SVM** |
| **Chen 2019** | **28/102** | **361** | **3-year Multicenter retrospective cohort study** | **Discriminate between P-MCI and N-MCI， and** **probability of MCI to AD** | **The ADNI, NADS study enrolled a total of 87 subjects with a baseline diagnosis of mild cognitive impairment (MCI) through community health screening, newspaper advertising, and hospital outpatient services.** | **NINCDS-ADRDA /Petersen/IWG** | **NR** | **NR** | **Keep continuous variables** | **10, three functional connectivity indices of the hippocampus, FCI, CC posterior cingulate cortex (PCCCC), Gray matter concentration index gmi, fusiform gyrus, Aβ1-42, p-tauMMSE, ADAS-Cog, and AVLT** | **NR** |
| **Chun 2022** | **254/705** | **462** | **3-year prospective cohort study** | **Identifying the probability of aMCI to AD** | **Conducted from June 2007 to December 2019 at Samsung Medical Center (SMC), South Korea, and followed for at least 3 years after baseline neuropsychological testing** | **DSM-IV/ evidence of cognitive impairment (confirmed by neuropsychological testing) and social and/or occupational dysfunction (confirmed by impairment in daily activities). /NINCDS-ADRDA** | **NR** | **LR** | **Keep continuous variables** | **19, age, sex, education, ApoE ε2, ApoE ε4, K-BNT, ideation and movement disorder, calculated total score, RCFT replication score, RCFT replication time, SVLT delayed memory, SVLT recognition score, RCFT delayed memory, RCFT recognition score, CP, go/no-go test, animal memory test, MMSE, and CDR-SOB** | **XGBoost** |
| **Velazquez 2021** | **39/383** | **245** | **5-year retrospective cohort study** | **Identifying patients MCI-C and MCI-NC， and** **probability of MCI to AD** | **ADNI-1、ADNI-2 and ADNI-GO** | **NR** | **Delete directly** | **RF** | **Keep continuous variables** | **9, ApoE4, hippocampal volume, ventricular volume, ADAS13, ADAS11, FAQ, MMSE, age, ethnicity** | **RF** |
| **Li 2014** | **NR/293** | **NR** | **4-year retrospective cohort study** | **Identifying patients MCI-C and MCI-NC， and** **probability of MCI to AD** | **ADNI** | **NR** | **NR** | **LASSO** | **NR** | **10, mean cortical thickness (CTA), standard deviation of cortical thickness (CTStd), cortical segmentation volume (Vol.Cord), volume of specific white matter regions (Vol.WM), total cortical surface area (Surf.A), 1, 4, 7 items in ADAS, FAQ, ApoE4** | **RF hierarchical interaction model** |
| **Kuang 2020** | **121/361** | **343** | **3-year prospective cohort study** | **Predicting the transition from MCI to AD** | **The Hongdu community (urban community), which has a large and stable elderly population in Nanchang City, China, was selected as the research site. We recruited seniors aged > 60 years and established a cohort of 1,913 people at the end of 2012** | **( i ) NIA–AA；( ii )Related Disorders Association Criteria (Revision) and the International Classification of Diseases;** | **NR** | **GB** | **converted to categorical variables** | **5, age, ADL score, urine AD7c - NTP, alcohol consumption, smoking** | **ANN** |

**ANN, Artificial neural networks; ADRDA=Alzheimer's Disease and Related Disorders Association (now the Alzheimer's Association) Work Group; IWG=International Working Group criteria; IWG–AA=International Working Group and Alzheimer's Association joint criteria.; NIA–AA=US National Institute on Aging and Alzheimer's Association joint criteria; NINCDS=US National Institute of Neurological and Communicative Disorders and Stroke criteria; RF: Random forests: ML,** **Machine learning; LR, logistic regression; KNN, k nearest neighbor; GBM, gradient boosting machine; LRP, layer-wise relevance propagation;** **ADL: Activity of daily living;** **MMSE: Mini-Mental State Examination; NR, not reported; XGBoost, eXtreme Gradient Boosting. AD, Alzheimer’s disease; ADAS-Cog, Alzheimer’s disease assessment scale-cognitive subscale; ADAS13, Modified ADAS-Cog 13-item scale; ADAS11, ADAS-Cog 11-item scale; ADNI , Alzheimer’s disease neuroimaging initiative; APOE4, Apolipoprotein E 4 allele; AUC, Area under the curve; CDR, Clinical dementia rating; CDR-SB, Clinical dementia rating sum of boxes; CSF, Cerebrospinal fluid; FAQ, Functional assessment questionnaire; HVI, Hippocampal volumetric integrity; LHVI, left hemisphere HVI; RHVI, right hemisphere HVI; MCI, Mild cognitive impairment; pMCI, progressive MCI subjects; sMCI, stable MCI subjects; MCI-C, MCI converter; MCI-NC, MCI non-converter; N-MCI， non-progressive MCI; p-tau:** **Highly phosphorylated tau protein; MRI, Magnetic resonance imaging ; GDS-15, Geriatric Depression Scale-15; SNP, single nucleotide polymorphism; PET, positron emission computed tomography ;** **LASSO: Least absolute shrinkage and selection operator; RCFT, Rey–Osterrieth Complex Figure Test; SVLT, Seoul Verbal Learning Test; CDR-SOB, Clinical dementia rating-sum of boxes;** **MRMR: Max-Relevance and Min-Redundancy; BSR, Best Subset regression. * : indicates an interactive item.**

***** **Sample Size:** **Calculate the minimum required sample size with the tool R pmsampsize.**

**Table 7: Model Validation and Performance**

| **Study** | **Validation Method** | **internal validation** | | | | | | **External Validation** | **Presentation** |
| --- | --- | --- | --- | --- | --- | --- | --- | --- | --- |
|  |  | **Discrimination AUC（95%CI）** | **Calibration** | **Sensitivity** | **Specificity** | **Accuracy** | **Other** |  |  |
| **Lee 2014** | **Internal** | **0.71(0.68–0.75)** | **Calibration curve** | **NR** | **NR** | **NR** | **NR** | **NR** | **Grading system** |
| **Grassi 2019** | **Bootstrap** | **0.88 (0.85–0.91)** | **NR** | **0.77** | **0.79** | **0.79** | **PPV:0.68, NPV:0.86,**  **F1:0.72** | **NR** | **Nomogram** |
| **Mubeen 2017** | **Nested cross-validation** | **0.87** | **NR** | **0.79** | **0.81** | **0.8** | **Confusion matrix** | **NR** | **NR** |
| **Maurik 2019** | **10-fold cross-validation、Out-of-bag（OOB)** | **0.74(0.71–0.76）** | **Calibration curve** | **NR** | **NR** | **NR** | **NR** | **NR** | **spread­ sheet calculator, APP** |
| **Bucholc 2023** | **5-fold cross-validation** | **0.6** | **NR** | **0.85** | **0.25** | **0.85** | **NR** | **NR** | **NR** |
| **Zhao 2022** | **10-fold cross-validation、RF-RFE** | **0.99** | **NR** | **1** | **0.95** | **0.97** | **F1:0.77** | **NR** | **NR** |
| **Mallo 2019** | **10-fold cross-validation** | **0.85** | **Calibration curve** | **0.6** | **0.69** | **0.88** | **F1:0.67** | **NR** | **Grading system** |
| **Lee 2019** | **5-fold cross-validation** | **0.86** | **Calibration curve** | **0.82** | **0.81** | **NR** | **F1:0.8** | **NR** | **Grading system** |
| **Li 2021** | **5-fold cross-validation** | **0.83** | **NR** | **0.71** | **0.78** | **0.79** | **NR** | **AUC of 0.78 (76.00% accuracy, 77.08% sensitivity, 75.32% specificity).** | **Equation formulas** |
| **Hojjati 2017** | **5-fold cross-validation** | **0.95** | **NR** | **0.83** | **0.9** | **0.91** | **NR** | **NR** | **NR** |
| **Korolev 2016** | **9-fold cross-validation** | **0.87** | **Calibration curve** | **0.83** | **0.76** | **NR** | **NR** | **NR** | **NR** |
| **Chen 2019** | **Nested cross-validation** | **0.8** | **Calibration curve** | **0.75** | **0.82** | **0.87** | **NR** | **AUC:85.71% ,Sensitivity:81.3% ，specificity:87.5%** | **Grading system** |
| **Chun 2022** | **Nested cross-validation** | **0.852** | **Calibration curve** | **NR** | **NR** | **0.807** | **NR** | **NR** | **Grading system** |
| **Velazquez 2021** | **5-fold cross-validation** | **0.992** | **Calibration curve** | **0.99** | **0.98** | **0.98** | **NR** | **NR** | **Graphical score sheets** |
| **Li 2014** | **holdout** | **NR** | **NR** | **0.82** | **0.75** | **0.89** | **NR** | **NR** | **Grading system** |
| **Kuang 2020** | **10-fold cross-validation** | **0.92** | **NR** | **0.75** | **0.66** | **0.81** | **PPV:0.75** | **NR** | **NR** |

**AUC, area under the receiver operating characteristic curve; NPV, negative predictive value; NR, not reported; PPV, positive predictive value; RFE, recursive feature elimination**

***If multiple models are presented, the values for the best performing model are reported.**

**Table 8:** **Assessment of risk of bias and concerns regarding applicability**

| **1.Participants** | **2.Predictors** | **3.Outcome** | **4.Analysis** |
| --- | --- | --- | --- |
| **Signalling questions** |  |  |  |
| 1.1. Were appropriate data sources used, e.g., cohort, RCT, or nested case-control study data? | 2.1. Were predictors defined and assessed in a similar way for all participants? | 3.1. Was the outcome determined appropriately? | 4.1. Were there a reasonable number of participants with the outcome? |
| 1.2. Were all inclusions and  exclusions of participants  appropriate? | 2.2. Were predictor assessments made without knowledge of outcome data? | 3.2. Was a prespecified or standard outcome definition used? | 4.2. Were continuous and categorical predictors handled appropriately? |
| - | 2.3. Were all predictors available at the time the model is intended to be used? | 3.3. Were predictors excluded from the outcome definition? | 4.3. Were all enrolled participants included in the analysis? |
| - | - | 3.4. Was the outcome defined and determined in a similar way for all participants? | 4.4. Were participants with missing data handled appropriately? |
| - | - | 3.5. Was the outcome determined without knowledge of predictor information? | 4.5. Was selection of predictors based on univariable analysis avoided? |
| - | - | 3.6. Was the time interval between predictor assessment and outcome determination appropriate? | 4.6. Were complexities in the data (e.g., censoring, competing risks, sampling of control participants) accounted for appropriately? |
| - | - | - | 4.7. Were relevant model performance measures evaluated appropriately? |
| - | - | - | 4.8. Were model overfitting, underfitting, and optimism in model-performance accounted for?** |
| - | - | - | 4.9. Did predictors and their assigned weights in the final model correspond to the results from the reported multivariable |
| **ROB** |  |  | analysis?** |
| Selection of participants | Predictors or their assessment | Outcome or its determination | Analysis |
| **Applicability** |  |  |  |
| Included participants or setting does not match the review question | Definition, assessment, or timing of predictors does not match the review question | Its definition, timing, or determination does not match the review question | - |

RCT = randomized controlled trial; ROB= risk of bias.

*For further details, please see the explanation and elaboration document, available at Annals.org. and www.probast.org. Signalling questions are answered as yes, probably yes, probably no, no, or no information. ROB and concerns for applicability are rated as low, high, or unclear.

**Development studies only.

**Table 9: Risk of bias and applicability assessment for each PROBAST domain**

| **study** | **Study (cohort)** | **ROB Participants** | **ROB Predictors** | **ROB Outcome** | **ROB Analysis** | **Applicability Participants** | **Applicability Predictors** | **Applicability Outcomes** | **ROB Overall** | **Overall Applicability** |
| --- | --- | --- | --- | --- | --- | --- | --- | --- | --- | --- |
| **Lee 2014** | **D** | **L** | **L** | **L** | **U** | **U** | **L** | **L** | **H** | **U** |
| **Grassi 2019** | **D** | **H** | **L** | **U** | **L** | **L** | **L** | **L** | **H** | **L** |
| **Mubeen 2017** | **D** | **L** | **L** | **U** | **U** | **U** | **L** | **L** | **H** | **U** |
| **Maurik 2019** | **D** | **L** | **L** | **U** | **L** | **L** | **L** | **L** | **H** | **L** |
| **Chun 2022** | **D** | **L** | **L** | **U** | **U** | **L** | **L** | **L** | **U** | **L** |
| **Bucholc 2023** | **D** | **L** | **L** | **L** | **H** | **L** | **L** | **H** | **H** | **H** |
| **Zhao 2022** | **D** | **L** | **L** | **U** | **H** | **L** | **L** | **L** | **H** | **L** |
| **Mallo 2019** | **D** | **H** | **L** | **U** | **H** | **L** | **L** | **H** | **H** | **H** |
| **Lee 2019** | **D** | **L** | **L** | **U** | **U** | **L** | **L** | **L** | **H** | **U** |
| **Li 2021** | **D** | **L** | **L** | **U** | **U** | **L** | **L** | **L** | **U** | **L** |
| **Hojjati 2017** | **D** | **L** | **L** | **U** | **H** | **U** | **L** | **L** | **H** | **U** |
| **Korolev 2016** | **D** | **L** | **L** | **U** | **H** | **U** | **L** | **L** | **H** | **U** |
| **Chen 2019** | **D** | **L** | **L** | **L** | **H** | **L** | **L** | **L** | **H** | **L** |
| **Velazquez 2021** | **D** | **L** | **L** | **U** | **U** | **L** | **L** | **L** | **H** | **L** |
| **Li 2014** | **D** | **L** | **L** | **U** | **U** | **U** | **L** | **L** | **H** | **U** |
| **Kuang 2020** | **D** | **L** | **L** | **L** | **U** | **L** | **L** | **L** | **H** | **L** |

***L: indicates low ROB/low concern regarding applicability; U: indicates unclear.ROB/unclear concern regarding applicability.; H: indicates high ROB/high concern regarding applicability. D: Development Model.**

**Fig.1: Prediction model Risk Of Bias assessment Tool (PROBAST) risk of bias assessment for all included models (N =16).**

**
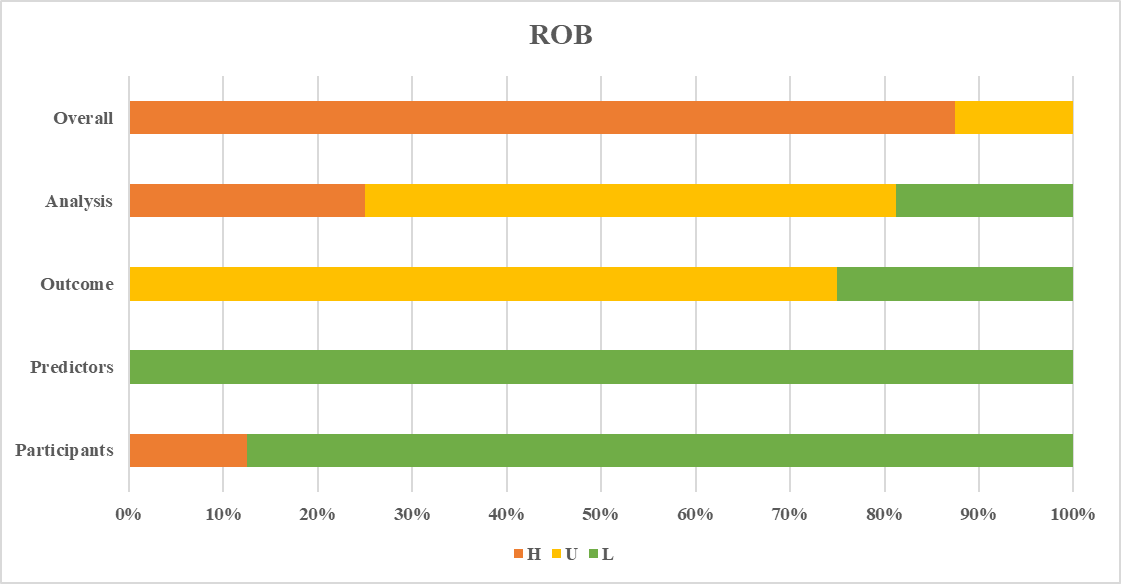
**

**Fig.2: Prediction model Applicability assessment Tool (PROBAST) risk of bias assessment for all included models (N =16).**

**
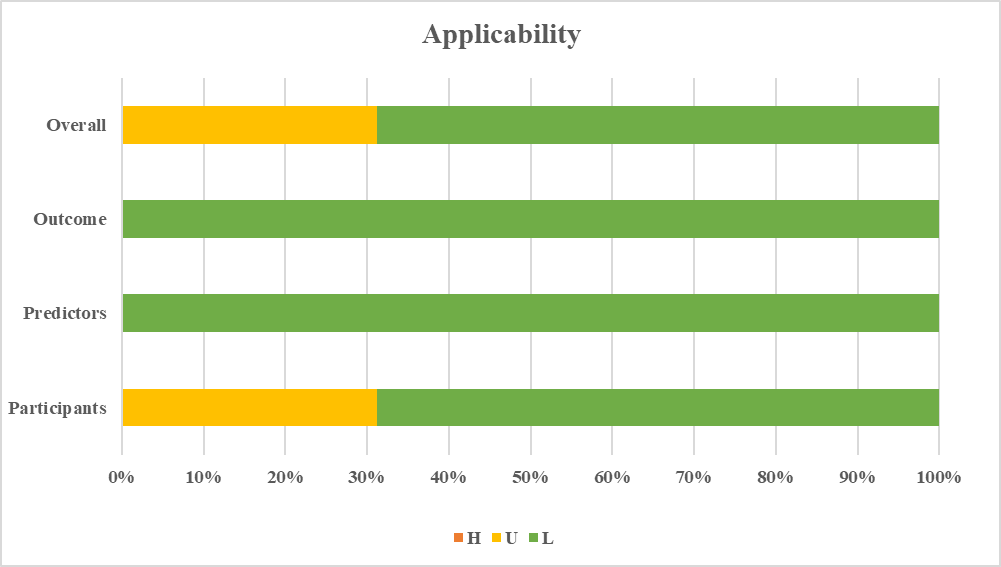
**

**Fig3: Funnel plot of all models included in primary meta-analysis**

**
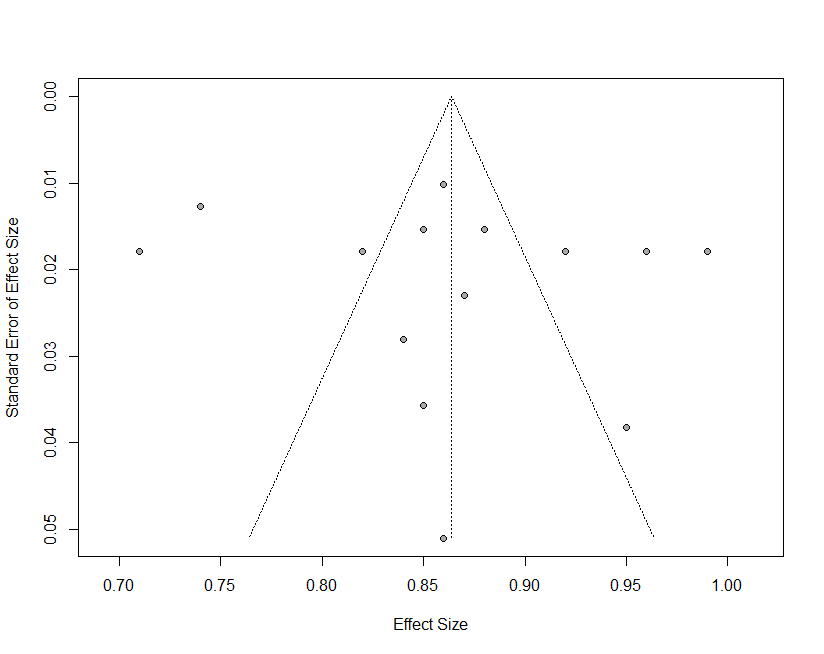
**

**Table 10: TRIPOD-SRMA Checklist for reporting systematic reviews of prediction model studies**

| **Section and topic** | **Item No** | **Checklist item** | **Page** |
| --- | --- | --- | --- |
| **Title** |  |  |  |
| **Title** | **1** | **Identify the report as a systematic review or meta-analysis (or both) of diagnostic or prognostic model studies. Specify the target population and outcome(s) predicted as relevant to the review question.** | **P1** |
| **Abstract** |  |  |  |
| **Abstract** | **2** | **See the TRIPOD-SRMA Checklist for Abstracts** | **P1-2** |
| **Introduction** |  |  |  |
| **Rationale** | **3** | **Describe the rationale for the review in the context of existing knowledge.** | **P2-3** |
| **Objectives** | **4** | **Provide an explicit statement of the objective(s) being addressed with reference to: target population, index and comparator models (as relevant), outcome(s), time (prediction horizon and intended moment of using the model), and setting.** | **Table 1** |
| **Methods** |  |  |  |
| **Study eligibility criteria** | **5** | **Specify study characteristics used as eligibility criteria, including any prediction models of specific interest, and whether development or validation studies (or both) were eligible.** | **P5** |
| **Information sources** | **6** | **Specify all databases, registers, websites, organisations, reference lists and other sources searched or consulted to identify studies. Specify the date when each source was last searched or consulted.** | **P4** |
| **Search strategy** | **7** | **Present the full search strategies for all databases, registers and websites, including any filters and limits used.** | **Table 1,** **Appendix File 1** |
| **Study selection process** | **8** | **Specify the methods used to decide whether a study met the inclusion criteria of the review, including how many reviewers screened each record and each report retrieved, whether they worked independently, and if applicable, details of automation tools used in the process.** | **P6-9,**  **Fig. 1** |
| **Data collection process** | **9** | **Specify the methods used to collect data from study reports, including how many reviewers collected data from each report, whether they worked independently, any processes for obtaining or confirming data from study investigators, and if applicable, details of automation tools used in the process.** | **P5** |
| **Data Items** | **10a** | **List and define all items for which data were sought from each study.** | **P6-7** |
|  | **10b** | **State the model performance measures that were sought (e.g., measures of calibration, discrimination, overall model fit, clinical utility).** | **P6-7** |
|  | **10c** | **Describe how any desired but unreported data items (items 10a, 10b) were handled (e.g., contacted authors, calculated from other reported information).** | **P7-8** |
| **Risk of bias and applicability assessment** | **11** | **Specify the methods used to assess risk of bias in the included studies and their applicability to the review question. This should be done separately for each model development and validation. Include details of any tool(s) used, how many reviewers assessed each study and whether they worked independently.** | **P7-8**  **Appendix Table 8** |
| **Synthesis methods** | **12a** | **Describe any methods for synthesising estimates of performance measures for each model. If meta-analysis was carried out, describe the methods used, including any transformations of data prior to pooling, how any heterogeneity in model performance was quantified and handled, and software package(s) used.** | **P8** |
|  | **12b** | **Describe any methods used to explore possible causes of heterogeneity in model performance (e.g., subgroup analysis, meta-regression), including whether or not they were planned.** | **P8** |
| **Section and topic** | **Item No** | **Checklist item** | **Page** |
| **Certainty assessment** | **12c** | **Describe any sensitivity analyses conducted to assess robustness of the synthesised results.** | **P8** |
|  | **13** | **Describe any methods used to assess certainty (or confidence) in the body of evidence for a prediction model.** | **P8** |
| **Results** |  |  | **Page** |
| **Study selection** | **14** | **Describe the results of the search and selection process, from the number of records identified in the search to the number of studies and models included in the review, ideally using a flow diagram.** | **P8, Fig. 1** |
| **Study and model**  **characteristics** | **15** | **Present study characteristics and model details extracted (as per Item 10a), and cite the study reports.** | **P8-13** |
| **Risk of bias and applicability** | **16** | **Present results of risk of bias and applicability assessment. This should be done separately for each model development and validation in each included study.** | **P12-14**  **Appendix Table 9, Fig.1, Fig.2** |
| **Results of model performance in individual studies** | **17** | **Present performance estimates and confidence intervals for each model and all evaluations, including whether they relate to the internal or external validation performance. If internal, give details of the method.** | **P11-12**  **Appendix**  **Table 6, 7** |
| **Results of syntheses** | **18a** | **Present the results of any synthesis of model performance, together with details of which study estimates contributed. If meta-analysis was carried out, then for each model and performance measure, present summary results, confidence/credible intervals and measures of heterogeneity. Forest plots may be useful.** | **Fig. 3 Fig. 4**  **P14-15, Appendix**  **Table 7** |
|  | **18b** | **For each model, present results of all investigations of possible causes of heterogeneity in model performance.** | **Fig. 3 Fig. 4**  **P13-14** |
|  | **18c** | **Present results of all sensitivity analyses conducted to assess the robustness of the synthesised results.** | **P13,**  **Appendix**  **Fig. 3** |
| **Certainty of evidence** | **19** | **Present any assessments of certainty (or confidence) in the body of evidence for each prediction model of interest.** | **P12-13,**  **Fig. 3 Fig. 4** |
| **Discussion** |  |  | **Page** |
| **Summary of evidence** | **20** | **Summarise the main findings including the strengths and limitations of the evidence.** | **P14-18** |
| **Limitations** | **21** | **Discuss the strengths and limitations of the review process.** | **P18** |
| **Implications** | **22** | **Discuss implications of the results in the context of other evidence and for practice, policy, and future research.** | **P18-19** |
| **Other information** |  |  | **Page** |
| **Registration and protocol** | **23a** | **Provide registration information for the review, including register name and registration number, or state that the review was not registered.** | **P1** |
|  | **23b** | **Indicate where the review protocol can be accessed, or state that a protocol was not prepared.** | **P1** |
|  | **23c** | **Describe and explain any amendments to information provided at registration or in the protocol.** | **inapplicable** |
| **Support** | **24** | **Describe sources of financial or non-financial support for the review, and the role of the funders or sponsors in the review.** | **P20** |
| **Competing interests** | **25** | **Declare any competing interests of review authors.** | **P20** |
| **Section and topic** | **Item No** | **Checklist item** | **Page** |
| **Availability of data, code, and other materials** | **26** | **Report which of the following are publicly available and where they can be found: template data collection forms; data extracted from included studies; data used for all analyses; analytic code; any other materials used in the review.** | **P20** |

**Table 11: PRISMA ABSTRACT CHECKLIST**

| **Section and Topic** | **Item #** | **Checklist item** | **Reported (Yes/No)** |
| --- | --- | --- | --- |
| **TITLE** | | |  |
| **Title** | **1** | **Identify the report as a systematic review or meta-analysis (or both) of diagnostic or prognostic model studies. Specify the target population and outcome(s) predicted as relevant to the review question.** | **Yes** |
| **BACKGROUND** | | |  |
| **Objectives** | **2** | **Provide an explicit statement of the main objective(s) being addressed with reference to: target population, index and comparator models (as relevant), outcome(s), time (prediction horizon and intended moment of using the model), and setting.** | **Yes** |
| **METHODS** | | |  |
| **Eligibility criteria** | **3** | **Specify study characteristics used as eligibility criteria, including any prediction models of specific interest, and whether development or validation studies (or both) were eligible.** | **Yes** |
| **Information sources** | **4** | **Specify the information sources (e.g. databases, registers) used to identify studies and the date when each was last searched.** | **Yes** |
| **Risk of bias and applicability** | **5** | **Specify the methods used to assess risk of bias in the included studies.** | **Yes** |
| **Synthesis of results** | **6** | **Specify the methods used to synthesise performance measures for each model of interest.** | **Yes** |
| **RESULTS** | | |  |
| **Included studies** | **7** | **Give the total number of included studies and participants and summarise relevant characteristics of studies.** | **Yes** |
| **Results of syntheses** | **8** | **Present results for each of the main models of interest. If meta-analysis was used to synthesise study estimates of model performance, report the summary result and confidence/credible interval for each performance measure, together with the number of study estimates contributing.** | **Yes** |
| **DISCUSSION** | | |  |
| **Limitations of evidence** | **9** | **Provide a brief summary of the limitations of the evidence included in the review.** | **Yes** |
| **Interpretation** | **10** | **Provide a general interpretation of the results and important implications for research and practice.** | **Yes** |
| **OTHER** | | |  |
| **Funding** | **11** | **Specify the primary source of funding for the review.** | **Yes** |
| **Registration** | **12** | **Provide the register name and registration number.** | **Yes** |
